# Advances and challenges in gene therapy strategies for pediatric cancer: a comprehensive update

**DOI:** 10.3389/fmolb.2024.1382190

**Published:** 2024-05-21

**Authors:** Amir Kian Moaveni, Maryam Amiri, Behrouz Shademan, Arezoo Farhadi, Javad Behroozi, Alireza Nourazarian

**Affiliations:** ^1^ Pediatric Urology and Regenerative Medicine Research Center, Tehran University of Medical Sciences, Tehran, Iran; ^2^ Stem Cell Research Center, Tabriz University of Medical Sciences, Tabriz, Iran; ^3^ Department of Genetics and Molecular Medicine, School of Medicine, Zanjan University of Medical Sciences, Zanjan, Iran; ^4^ Department of Cell and Molecular Biology, Faculty of Biological Sciences, Kharazmi University, Tehran, Iran; ^5^ Department of Basic Medical Sciences, Khoy University of Medical Sciences, Khoy, Iran

**Keywords:** gene therapy, pediatric cancer, CRISPR-Cas9, gene editing, delivery systems

## Abstract

Pediatric cancers represent a tragic but also promising area for gene therapy. Although conventional treatments have improved survival rates, there is still a need for targeted and less toxic interventions. This article critically analyzes recent advances in gene therapy for pediatric malignancies and discusses the challenges that remain. We explore the innovative vectors and delivery systems that have emerged, such as adeno-associated viruses and non-viral platforms, which show promise in addressing the unique pathophysiology of pediatric tumors. Specifically, we examine the field of chimeric antigen receptor (CAR) T-cell therapies and their adaptation for solid tumors, which historically have been more challenging to treat than hematologic malignancies. We also discuss the genetic and epigenetic complexities inherent to pediatric cancers, such as tumor heterogeneity and the dynamic tumor microenvironment, which pose significant hurdles for gene therapy. Ethical considerations specific to pediatric populations, including consent and long-term follow-up, are also analyzed. Additionally, we scrutinize the translation of research from preclinical models that often fail to mimic pediatric cancer biology to the regulatory landscapes that can either support or hinder innovation. In summary, this article provides an up-to-date overview of gene therapy in pediatric oncology, highlighting both the rapid scientific progress and the substantial obstacles that need to be addressed. Through this lens, we propose a roadmap for future research that prioritizes the safety, efficacy, and complex ethical considerations involved in treating pediatric patients. Our ultimate goal is to move from incremental advancements to transformative therapies.

## 1 Introduction

Despite being less common worldwide, childhood cancer presents a unique set of challenges that differ from those of adult cancers (Agrawal and Bansal, 2019). Approximately 400,000 children and adolescents ages 0–19 are diagnosed with cancer annually (Organization, 2021). According to the World Health Organization, childhood cancer survival rates vary widely by geography. Children in high-income countries have a survival rate of over 80%, while survival rates in many low- and middle-income countries fall below 30% (Childhood Cancer, 2023). The unique biological characteristics and age-dependent dynamics of childhood cancer present significant barriers to detection, diagnosis, and treatment. These barriers often lead to long-lasting complications that can affect critical developmental aspects, highlighting the need for therapeutic strategies that optimize survival and mitigate adverse effects (Blattner-Johnson et al., 2022).

The molecular basis of pediatric cancer differs from many adult epithelial tumors, which exhibit high mutation rates in oncogenes and tumor suppressors (Kirches et al., 2021). Pediatric cancers generally have fewer mutations but a higher frequency of chromosomal structural rearrangements resulting in oncogenic gene fusions (Newman et al., 2021). An example is the ETV6-RUNX1 fusion gene resulting from a t (Jones et al., 2019; Yahya and Alqadhi, 2021) translocation that is associated with 25% of pediatric B-cell acute lymphoblastic leukemia (ALL) cases, a fusion gene that is absent in adult leukemias (Aydin et al., 2016). In the field of oncology, researchers have discovered important genetic alterations. One example is the t (Ruan et al., 2020; Martinez et al., 2022) BCR-ABL1 fusion, which is often found in cases of acute lymphoblastic leukemia (ALL) in children. Another example is the t (Laetsch et al., 2021; Martinez et al., 2022) EWSR1-FLI1 fusion, which is frequently linked to Ewing sarcoma. These specific chromosomal translocations play a vital role in the development of these types of cancers (Sole et al., 2021). The relative rarity of mutations may reflect the shorter time frame for accumulation of genetic defects early in life (Ruan et al., 2020). Nevertheless, certain consistent mutational hotspots are observed, such as activating mutations in the RAS-MAPK pathway in juvenile myelomonocytic leukemia (Kim et al., 2021). Table 1 shows the different types of pediatric cancers and their associated genetic alterations.

**TABLE 1:** Overview of pediatric cancer types and their genetic alterations.

Cancer type	Common genetic alterations	Prevalence in pediatric population	Reference
Brain tumors	KIAA1549-BRAF, BRAFV600E, FGFR1 mut or rearrangement, NF1, SMARCB1 (INI1), SMARCA4, microRNA cluster C19MC amplification, FOXR2 rearrangements, BCOR ITD, CTNNB1, DDX3X, SMARCA4, TP53, PTCH1, SUFU, SMO, GLI2, and TP53	20%–25% of pediatric cancer	Jones et al. (2019)
ALL	CDKN2A/B deletion	15%–35% of pediatric patients with *de novo* ALL	Martinez et al. (2022)
Retinoblastoma	RB1 tumor-suppressor gene, polymorphisms in p53, CDKN1A, and CDKN2A, genetic modifiers like MDM2, MDM4, or MED4	Approximately 40% of all cases of retinoblastoma are classified as hereditary, with the majority of those individuals having tumors present in both eyes	Capasso et al. (2020)
Wilms tumor	*WT1*, mutations in REST, CHEK2, and PALB2	Most (95%) of these tumors are diagnosed in children under 10 years old	Treger et al. (2019)
Neuroblastoma	ASCL1 and PHOX2B	8% of all malignancies in children	Guan et al. (2021)

ALL, acute lymphoblastic leukemia; CDKN, cyclin-dependent kinase inhibitor.

The unique mutational landscape and cytogenetic abnormalities of pediatric tumors provide excellent opportunities for targeted therapy, particularly gene therapy (Laetsch et al., 2021). Gene therapy—a promising approach to treating disease by altering the genetic content of patients’ cells—shows potential for greater efficacy and specificity than traditional chemotherapy, especially in pediatric cancers with identified genetic drivers (de Lartigue, 2018; Yahya and Alqadhi, 2021).

The immense potential of gene therapy in combating resistant pediatric malignancies has been demonstrated in recent preclinical studies and clinical trials (Campbell et al., 2020). For example, chimeric antigen receptor (CAR) T-cell therapy has shown high remission rates in relapsed B-cell ALL by modifying the patient’s own T cells to recognize and eradicate cancer cells (Voynova and Kovalovsky, 2021). Early phase trials of CAR T-cells have also shown promising results in pediatric solid tumors (Vitanza et al., 2021). Beyond immunotherapy, some studies have shown improved outcomes by directly correcting single gene defects (Labrosse et al., 2019).

However, as promising as gene therapy appears to be, it also faces several challenges that hinder its widespread clinical adoption. Efficient delivery to targeted tumor tissues, off-target genomic alterations, and potential adverse immune responses are major hurdles (Kirschner and Cathomen, 2020; Jeong et al., 2021). The high costs of personalized cell therapies, complex manufacturing requirements, and regulatory uncertainties regarding emerging genetic technologies are additional considerations (Narayanan, 2016). Therefore, systematic research efforts are crucial to address these limitations, improve safety profiles, demonstrate efficacy through rigorous trials, and develop innovative strategies for improved accessibility.

This review aims to provide a thorough assessment of the current landscape and future prospects of gene therapy in pediatric oncology. We will review the genetic mechanisms underlying major childhood cancers and outline recent preclinical and clinical advances in gene therapy for various malignancies. We will also examine key challenges such as delivery barriers, safety concerns, manufacturing issues, and potential solutions. The advancement of gene therapy approaches represents a significant opportunity to usher in an era of personalized, targeted medicine that can dramatically improve cure rates and quality of life for children with cancer worldwide. To realize this potential paradigm shift, collaborative efforts across research, medicine, industry, and government are needed to systematically overcome the challenges that impede clinical translation.

## 2 Overview of gene therapy in pediatric cancer

A seismic shift in pediatric cancer treatment has occurred with the advent of gene therapy. This medical revolution is based on the principle of altering the genetic makeup of cells to treat or prevent disease (Yahya and Alqadhi, 2021). Faulty genes can lead to the development of pediatric cancers, hence the promising strategy of using viral vectors to introduce functional copies (Hacker et al., 2020).

Gene therapy facilitates the construction of tailored, targeted therapies that depend on the unique genetic drivers of various pediatric malignancies (Johnson, 2018). For example, the introduction of tumor suppressor genes serves to counteract pro-growth mutations (Gregory and Copple, 2023). Survival rates in certain pediatric cancers have been significantly improved with gene therapy compared to standard therapy alone, according to recent studies (Hunger et al., 2012; Nowicki et al., 2016). Due to the lack of mutations from environmental exposures, pediatric tumors are well-suited for genetic approaches due to germline mutations (Kilburn and Packer, 2020).

**FIGURE 1:**
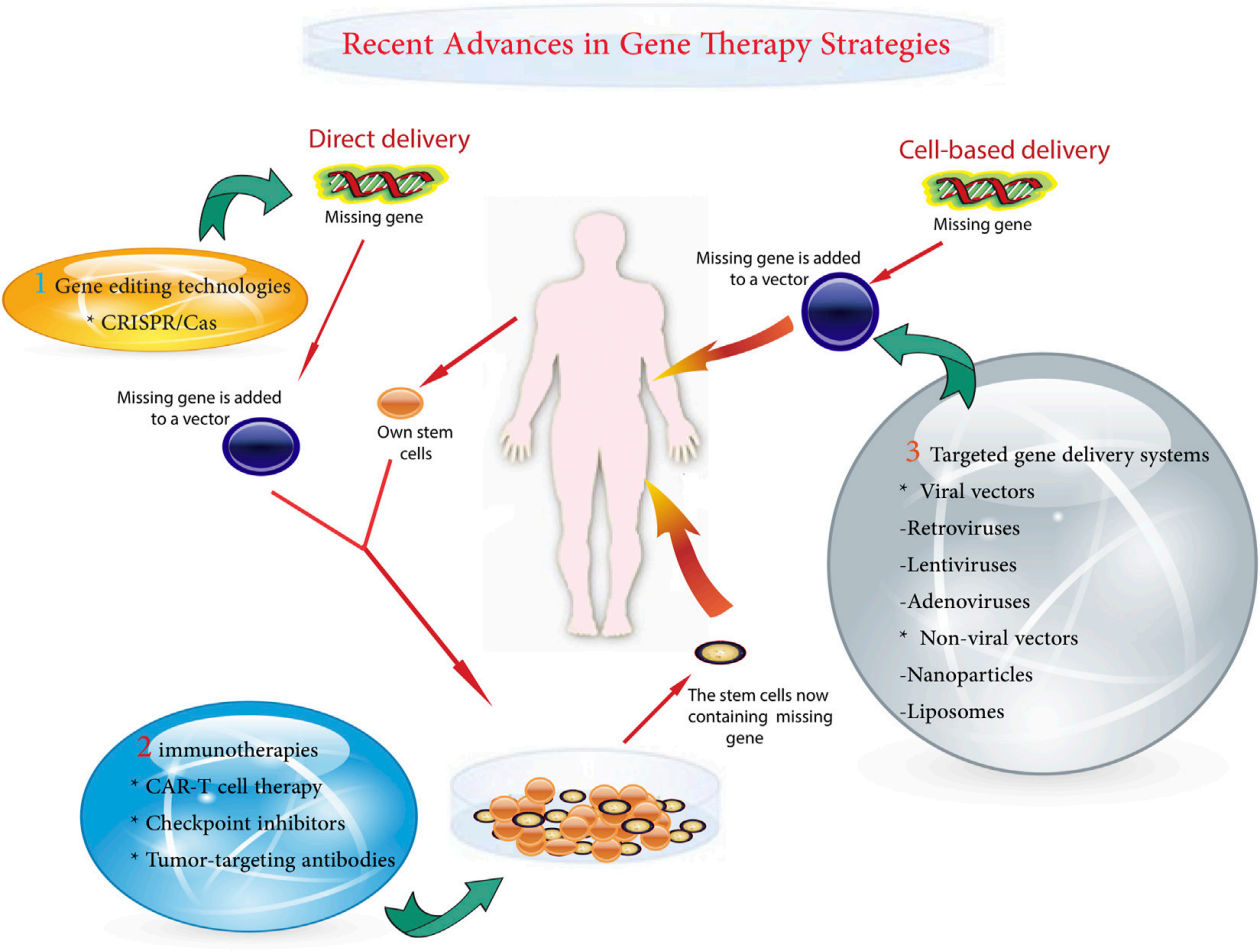
Schematic diagram of gene delivery methods.

A variety of gene therapy strategies are currently being used in pediatric oncology. These range from the correction of single gene defects to the delivery of suicide genes that selectively kill cancer cells (Das et al., 2015). Powerful gene-editing tools, such as clustered regularly interspaced short palindromic repeats (CRISPR)-Cas9, allow for precision targeting but require rigorous safety testing for off-target effects (Atkins et al., 2021; Guo et al., 2023). One of the major challenges is to ensure precise delivery of the therapeutic gene to cancer cells while preventing unintended modifications (Chen et al., 2018). Other obstacles include potential immunogenicity and regulation of the expression of the introduced genes (Ziegler et al., 2018). The main gentherapy metohds shown in Figure 1.

As gene therapy research continues to advance, the prospect of more effective and less toxic treatments continues to grow (Deverman et al., 2018). Despite existing challenges, gene therapy has immense potential to usher in a new era of personalized medicine for childhood cancer. Sustained research will be essential to overcome current obstacles and fully realize the innovative promise of genetic approaches. Major milestones in gene therapy for childhood cancer are outlined in Table 2.

**TABLE 2:** Timeline of major milestones in gene therapy for pediatric cancer.

Year	Milestone	Description	Reference
1990	First gene therapy clinical trial	The first gene therapy clinical trial was conducted on a patient with a rare genetic disease, marking the beginning of gene therapy research	Danaeifar (2022)
1992	Stem cells used as a vector to deliver therapeutic genes	The goal of this first trial conducted by Bordignon et al. was to correct SCID syndrome caused by a deficiency in ADA	Bordignon et al. (1995)
2000	Two French patients with SCID experienced gene therapy	Gene therapy has been successfully used by French researchers to treat SCID	Dobson (2000)
2003	First human trial of gene therapy using modified lentivirus as a vector	The vector used for this trial was based on human adenovirus type 5, deleted in E1 and E4, and contained human OTC cDNA	Raper et al. (2003)
2006	Genetically engineered lymphocytes used for cancer treatment	Adoptive transfer of these transduced cells in 15 patients resulted in durable engraftment at levels exceeding 10% of peripheral blood lymphocytes for at least 2 months after the infusion	Morgan et al. (2006)
2010	CAR T-cell therapy study performed on B-cell malignancies	Adoptive transfer of anti–CD19-CAR-expressing T cells is a promising new approach for treating B-cell malignancies. The prolonged elimination of CD19^+^ cells in this patient indicates *in vivo* antigen-specific activity of anti–CD19-CAR-expressing T cells	Kochenderfer et al. (2010)
2017 or 2018	The first gene therapy was approved in United States	This year, the FDA approved two pioneering treatments, Kymriah and Yescarta, that use a patient’s own immune cells to fight rare types of cancer	Ledford (2020)
2020	CRISPR treatment inserted directly into the body for first time	Gene editing leaps to the next level with the injection of a CRISPR complex directly into a patient’s eye to combat a form of hereditary blindness	Mahase (2021)
2021	NHS England agreed to a deal for gene therapy for spinal muscular atrophy	NHS England agreed to a deal to ensure that Zolgensma, a one-off gene therapy medicine, will be made available for patients with spinal muscular atrophy	Mahase (2021)
2022	Pediatric leukemia in remission after base-editing cancer treatment in UK	GOSH in the UK released details of its first-in-human (T-cell ALL) clinical trial using base-edited CAR T-cells	leukaemia (2022)
2022	The first allogeneic CAR T Phase 2 trial is initiated	Potentially pivotal Phase 2 clinical trial of ALLO-501A in patients with relapsed or refractory large B-cell lymphoma. ALLO-501A is TALEN-edited in a number of ways to mitigate the risk of GVHD	Smirnov et al. (2021)

### 2.1 Types of gene therapy approaches used in pediatric cancer treatment

#### 2.1.1 Gene replacement therapy

Gene replacement therapy has emerged as a central strategy in the field of gene therapy and has shown great promise in the treatment of certain pediatric cancers (Pearl et al., 2023). The thrust of this approach is to identify a particular mutated gene in a tumor and use viral vectors to deliver a normal, “wild type” version of that gene to the cancer cells (Bhattacharjee et al., 2022). The goal of introducing a functional copy of the aberrantly mutated gene is to normalize the cancer’s genetics and limit its proliferation (Pscherer et al., 2006).

This therapy is particularly suitable for cancers driven by single-gene mutations, such as the BCR-ABL1 translocations characteristic of pediatric ALL (Sugapriya et al., 2012). However, there are challenges in selecting the optimal vector for gene delivery. While viruses could effectively infect human cells, they can induce immune responses against the introduced gene, potentially limiting efficacy (Aledo-Serrano et al., 2021). Another challenge is to ensure sustained expression of the delivered gene.

Nevertheless, gene replacement therapy has been successful in certain pediatric cancers. For example, in ALL, the introduction of CAR genes into patients’ T cells has resulted in high remission rates in refractory disease (Wu et al., 2021). Early studies have shown promising results in using gene replacement to correct mutated MERTK genes in neuroblastoma. Vollrath et al. found that administering recombinant adenovirus encoding MERTK subretinally reversed the defect in retinal pigment epithelium phagocytosis and rescued photoreceptors from degeneration in juvenile rats. As a result, the treated areas in animal models, which already exhibited significant pathology, appeared nearly normal (Vollrath et al., 2001).

The potential of gene replacement therapy to restore conventional function by targeting single genetic drivers underscores its relevance for precision treatment of pediatric malignancies (Senft et al., 2017). Expansion of this promising approach will require refinement of gene delivery methods to improve safety and efficacy, long-term outcome studies, and expansion of trials across the spectrum of pediatric cancers driven by targetable mutations (Magnani et al., 2013). Overcoming existing barriers could pave the way for a new generation of highly specific cancer therapies that could potentially eradicate mutation-driven pediatric tumors.

#### 2.1.2 Gene suppression therapy

Gene suppression therapy, often referred to as gene silencing, symbolizes a breakthrough approach to gene therapy that aims to combat disease by silencing harmful genes responsible for disease development. This method of silencing deleterious genes appears promising in the treatment of cancers activated by gain-of-function mutations, particularly those associated with pediatric malignancies (He and Jia, 2020).

RNA interference (RNAi), a powerful tool for gene silencing, inhibits the translation of targeted mRNAs via complementary base pairing. This is generally accomplished by the incorporation of either synthetic siRNAs or vector-derived shRNAs (Li et al., 2018). This approach has potential advantages in the treatment of pediatric cancers. For example, neuroblastoma cells harboring anaplastic lymphoma kinase (ALK) mutations experienced growth inhibition in experimental models following ALK silencing by RNAi (Schulte and Eggert, 2021). Thus, the approach of silencing oncogenic ALK could develop beneficial therapies for patients with this particular abnormality (Ji et al., 2021). Furthermore, RNAi-mediated targeting of beta-catenin in hepatoblastoma models resulted in tumor growth suppression, suggesting another potential target (Indersie et al., 2017). However, the challenge is to deliver RNAi triggers specifically to the tumor tissue. MicroRNAs (miRNAs), another regulatory approach, are short non-coding RNAs that reduce protein production by binding to mRNAs (Arraiano, 2021). A positive correlation was observed between miRNAs expression and tumor grade and type, with the highest expression noted in medulloblastomas, followed by ependymomas, and the lowest in pilocytic astrocytomas. Upregulation was predominantly observed in most members of the miR-17–92, miR-106a-363, and miR-106b-25 clusters, with miR-18a and miR-18b exhibiting the highest expression. Other miRNAs such as miR-19a, miR-92a, miR-106a, miR-93, and miR-25 also demonstrated elevated values. Additionally, miR-17-5p and miR-20a displayed high expression levels in medulloblastomas and ependymomas while approaching control levels in pilocytic astrocytoma samples. Notably, miRNA expression was also influenced by tumor grade and histology in pediatric tumorigenesis, suggesting therapeutic potential for miRNA (Gruszka et al., 2021). Measures such as adjusting abnormal miRNA levels or using miRNAs to suppress fusion oncogenic transcripts could be employed (Gambari et al., 2016). For example, chemotherapy showed higher efficacy in neuroblastoma cells where let-7 miRNA levels were restored (J et al., 2015). Nevertheless, the development of safe and effective miRNA-based therapies has encountered many obstacles, such as stability, off-target effects, and efficient delivery to target tissues (Roy et al., 2022).

Targeted delivery of gene silencing therapies to malignant cells is a challenging task (Safarzadeh Kozani et al., 2021a). Systemic administration may induce off-target gene silencing and consequent toxicity. Therefore, innovative delivery systems are being explored to increase specificity and avoid activation of immune responses (Zhao et al., 2020a). Studies are considering the use of bioengineered viruses, liposomes, and nanoparticles loaded with ligands for receptors overexpressed on tumor cells (He et al., 2021). However, other challenges remain, such as transient gene silencing and variable silencing efficacy due to accessibility of the target site (Safari et al., 2017).

In summary, despite the obstacles, gene silencing methods could play a critical role in accurately targeting and silencing genes that promote pediatric cancer growth (Verreault et al., 2006). In summary, the advent of paradigm-shifting gene editing technologies such as CRISPR and transcription activator-like effector nucleases (TALENs) has invigorated genetic research by providing customizable, targeted genome editing capabilities. However, with the tremendous opportunity comes the responsibility to guide the appropriate use of these rapidly advancing tools that hold the power to permanently recode life. Realizing their potential while mitigating the risks will require ongoing ethical discussions and flexible regulations based on scientific evidence. The coming decade promises exciting advances in gene editing, but it also demands caution.

## 3 Advantages and limitations of gene therapy in the context of pediatric cancer treatment

Breakthrough gene editing technologies such as CRISPR/Cas9 and TALENs have advanced genetic engineering and molecular biology research by facilitating precise genome editing (Noel et al., 2021). The CRISPR/Cas9 system, derived from the bacterial immune system, consists of the Cas9 enzyme that cleaves DNA and a guide RNA that targets the precise DNA sequence for modification. This mechanism allows for versatile genome manipulation by deleting, adding, or modifying specific DNA sequences (Aslam et al., 2021). Conversely, TALENs are engineered enzymes designed to bind and cleave desired DNA sites, providing an alternative precise genome editing approach (Becker and Boch, 2021). These innovative techniques have expanded opportunities in gene therapy, agriculture, and the understanding of genetics and disease (Libutti, 2014).

CRISPR/Cas9 techniques have undergone significant refinement, increasing the efficiency, specificity, and adaptability of genome editing (Schreurs et al., 2021). A prominent development in this area is prime editing, a sophisticated CRISPR technique that introduces precise DNA edits without the need for double-strand breaks or donor templates, instead inscribing new genetic information directly into target sites (Hao et al., 2021). This increases the range of potential edits while minimizing unexpected mutations. Similarly, TALEN methods have undergone significant improvements in design, assembly, and delivery, resulting in improved precision of genetic modifications (Sakuma et al., 2013). Emerging technology platforms now facilitate automated, high-throughput assembly of customized TALENs (Zhang et al., 2013). In addition, the combined use of TALENs and CRISPR technologies promotes a synergistic effect that optimizes precision and streamlines the editing process (Xu et al., 2015).

In the field of medicine, CRISPR and TALENs have the potential to precisely edit the human genome to treat genetic diseases by permanently modifying disease-causing mutations (Xu et al., 2015; Kesavan, 2023). An example of this has been reported in the treatment of sickle cell disease by editing hematopoietic stem cells (Anurogo et al., 2021). However, further improvements in safety and efficacy are needed before these techniques meet widespread clinical application (Anurogo et al., 2021). In agriculture, gene editing has been used to create pest-resistant crops and healthier livestock, a result touted to improve food security (Karavolias et al., 2021). However, discussions about regulations and appropriate applications continue. CRISPR and TALENs have become critical research tools for genetic screening, disease modeling, and elucidation of gene function in cellular and animal models (Zhao et al., 2016).

The continued development of these impressive tools has ethical, legal, and social implications that need to be addressed (Zhao et al., 2016). Concerns have been raised about unintended changes induced by CRISPR that deviate from the target gene, with potentially far-reaching consequences. In addition, the notion of human germline editing is controversial due to the potential risk of permanent, heritable changes (de Wert, 2019). For this reason, an ongoing dialogue among researchers, policymakers, and the public is essential to establish a framework that balances benefits and risks. Although gene editing has immense potential to combat disease, improve food security, and deepen genetic understanding, it must be approached cautiously because it involves permanent and extensive changes to living systems. Regulatory policies must evolve with scientific progress to guide responsible use (Rocha et al., 2020).

In summary, transformative gene editing technologies like CRISPR and TALENs have supercharged genetics research through customizable, targeted genome editing capabilities. But along with the immense opportunities come responsibilities to guide the appropriate use of these rapidly advancing tools that can permanently alter the code of life. Harnessing their potential while mitigating risks will require ongoing ethical debates and evolving regulations rooted in scientific evidence. The next decade of gene editing promises exciting progress but demands prudence in equal measure.

### 3.1 Recent advances in gene therapy strategies for pediatric cancer

The critical role of targeted gene delivery in gene therapy is undeniable, particularly in realizing the potential to redefine the treatment of genetic diseases (Pan et al., 2021). Among the tools at our disposal for this purpose are adeno-associated viruses (AAVs), which have shown impressive efficacy and specificity in delivering therapeutic genes to target cells (Tustian and Bak, 2021). These are not just viruses; they have been engineered to maximize delivery, with new AAV variants engineered through capsid engineering to improve targeting capabilities while minimizing the immune responses triggered by their natural counterparts (Lugin et al., 2020). For example, an optimized AAV was recently used in a study to deliver CRISPR to the liver of a mouse, demonstrating high specificity. AAVs have already found applications in clinical trials for conditions such as inherited retinal diseases, spinal muscular atrophy, and hemophilia (Moscoso and Steer, 2019).

There are also non-viral delivery systems in play, including lipid nanoparticles (LNPs) and polymeric nanoparticles, which have distinct advantages over viral vectors, such as a streamlined production process, reduced immunogenicity, and the ability to carry larger genetic cargo (Torres-Vanegas et al., 2021; Zohri et al., 2021). The mRNA COVID-19 vaccines made possible by LNPs illustrate their potential in gene therapy applications. Meanwhile, polymeric nanoparticles have achieved preclinical success in carrying CRISPR/Cas9 for gene editing as a possible therapy for genetic diseases (Ahn et al., 2021; Andresen and Fenton, 2021).

However, we must continue to refine vectors and nanoparticles to improve delivery efficiency and specificity while eliminating toxicity. There is potential for synergistic effects when viral and non-viral approaches are combined (Ren et al., 2021). For example, LNPs could potentially attenuate the inflammation induced by viral vectors. The importance of advancing gene delivery technology cannot be overstated, as we aim to unleash the full power of gene therapy in a range of applications, from genetic diseases to vaccines (Piperno et al., 2021).

Gene therapy has already produced groundbreaking treatments for certain inherited diseases; however, delivery has proven to be a limiting factor in progress (Michalakis et al., 2021). The advent of a new generation of customized viral vectors and non-viral systems provides optimism that these hurdles can be overcome, paving the way for a range of innovative gene therapies. It is critical that we maintain rigorous standards of development and ethical use as these powerful technologies are developed and applied (Ghosh et al., 2020).

In summary, the prospects for targeted gene therapy are being reshaped by the emergence of customized viral vectors and non-viral nanoparticle systems. Further modifications and ethical oversight are required to ensure the best therapeutic outcomes. The possibility of synergistic effects with the combination of viral and non-viral methods appears promising. Advances in gene therapy delivery methods offer hope for precision gene therapies, provided that progress is guided by the responsibility to maintain safety and ethical standards.

#### 3.1.1 Viral vectors (retroviruses, lentiviruses, adenoviruses, etc.)

Viral vectors have gained significant attention in gene therapy, as they have been transformed into useful gene delivery mechanisms due to their inherent ability to deliver genetic material into host cells. The main variants used in this context are retroviruses, lentiviruses, and adenoviruses (Giacca and Zacchigna, 2012).

Retroviruses, being RNA viruses, undergo a reverse transcription process in which their RNA genome is converted to DNA and further integrated into the host genome, facilitating persistent and stable gene expression within the host cell. The potential tumorigenesis induced by insertional mutagenesis is a notable risk (Steckbeck et al., 2014; Sabatino et al., 2022). Indeed, retroviruses exhibit efficient infection properties toward dividing cells—an aspect that is critical for their application in *ex vivo* treatments, as in the case of CAR T-cell therapy for leukemia (Heyman et al., 2022).

Similar to retroviruses, lentiviruses can affect both dividing and non-dividing cells, expanding their potential therapeutic applications, including but not limited to neuroscience and human immunodeficiency virus (HIV) treatment (Fassati, 2006). One of their notable advantages is their broad cell tropism, which allows them to transduce multiple cell type (Rainey and Coffin, 2006; Poletti and Mavilio, 2018). However, the risk of insertional mutagenesis associated with lentiviruses remains significant (Woods et al., 2003). The class of adenoviruses, which are double-stranded DNA viruses that do not integrate into the host genome, greatly reduces the risk of mutagenesis (Charman et al., 2019). Their efficacy in delivering transgenes to a variety of tissues *in vivo* and *ex vivo* is complemented by their large transgene capacity (Morrissey et al., 2012). They have shown impressive results in cancer gene therapy, enabling localized delivery of tumor suppressor genes (Wang et al., 2010). Their efficacy may be limited in certain therapeutic settings due to anti-adenovirus immune responses (Zhou et al., 2020).

Significant progress has been made in refining viral vector design and production methods, which have greatly improved safety and delivery efficiency (Li et al., 2021). An example is the advent of self-inactivating vectors that reduce the oncogenic risks associated with retroviruses and lentiviruses. Similarly, third-generation adenoviral vectors are designed to minimize unwanted immune activation (Troyanovsky et al., 2015; Shaw and Suzuki, 2019).

In summary, despite their unique profiles, retroviral, lentiviral, and adenoviral vectors have been instrumental in the advancement of gene therapy. With continued improvements in viral vector biology and engineering, their medical utility is expected to expand. A better understanding of cell-specific targeting may lead to vectors with superior therapeutic precision in the future.

#### 3.1.2 Non-viral vectors (liposomes, nanoparticles, etc.)

Due to the various challenges associated with viral vectors, such as immunogenicity risk, limited payload capacity, and high production costs (Luis, 2020), alternative non-viral vectors for gene delivery have come to the forefront. These alternatives, which include liposomes, nanoparticles, and others, have their own unique advantages and specific issues that need to be addressed (Musale and Giram, 2021).

Liposomes, spherical vesicles composed of lipid bilayers, have been identified as safer alternatives capable of encapsulating and delivering genes, proteins, or drugs with the added benefit of minimizing immunogenic responses or infections (Liu et al., 2022; van der Koog et al., 2022). One of the identified problems with liposomes is that they often exhibit encapsulation efficiencies below 20% (Costa et al., 2021), and there is a constant risk of rapid degradation of the cargo before it reaches its intended target (Eloy et al., 2014).

To address these limitations, cationic liposomes have been developed to enhance encapsulation by facilitating the formation of complexes with negatively charged nucleic acids (Ewert et al., 2021). In addition, surface modifications, such as the attachment of antibodies, peptides, or saccharides, can ensure that target specificity is enabled (Lestini et al., 2002). For example, anti-EGFR nanobodies have been attached to liposomes to facilitate targeted drug delivery to colon cancer cells (Narbona et al., 2023). Further refinement of the size, composition, and surface charge of these liposomes aims to improve stability, efficiency, and cellular uptake (Ma et al., 2013). Nanoparticles represent a diverse range of non-viral vectors incorporating polymers, lipids, metals, or other elements (Botto et al., 2018). For example, gold nanoparticles provide controlled, sustained release of genes, making them invaluable in the treatment of solid tumors (Zhao and Liu, 2014). Other nanoparticle vectors include polymeric nanoparticles, quantum dots, and liposomal nanoparticles (Ishihara et al., 2011). Polyplexes, a type of nanoparticle vector, are widely used due to their ease of production, safety, customization potential, and ability to encapsulate various nucleic acids (Bai et al., 2014). Despite these advantages, they still present challenges such as short circulation time, low transfection efficiency, and cytotoxicity (Ita, 2020).

LNPs have shown potential for mRNA delivery, as demonstrated by the use of mRNA COVID-19 vaccines (Melamed et al., 2022). With high encapsulation efficiency, biocompatibility, and stability, LNPs can be functionalized for targeting (Alipour et al., 2017). To realize their full potential, new surface modifications are being explored to increase stability, reduce off-target effects, improve specificity, and decrease toxicity (Kulkarni and Feng, 2011).

In conclusion, replacement non-viral vectors such as liposomes and nanoparticles offer versatility that is stimulating research aimed at overcoming current limitations. As safer alternatives to viral vectors with the potential for significant optimization, they could greatly improve gene therapy and drug delivery methods. Looking ahead, the future seems bright for non-viral vectors as newer designs, modifications, and combinations continue to emerge.

### 3.2 Immune-based gene therapies

#### 3.2.1 CAR T-cell therapy

CAR T-cell therapy is a revolutionary approach to cancer treatment that differs significantly from previous immunotherapy techniques. This innovative therapy uses a patient’s own lymphocytes, commonly referred to as T-cells, to identify and eradicate cancer cells with extreme precision (Xu et al., 2021). The key steps in CAR T-cell therapy involve the isolation of a patient’s T cells, followed by genetic modification using viral vectors to create CARs, their subsequent expansion *in vitro*, and finally their reintroduction into the patient’s body (Ke et al., 2021). These engineered CARs can bind to target antigens on cancer cells with an affinity in the nanomolar range, 100 to 1,000 times stronger than natural antibodies (Harris and Kranz, 2016). This binding induces cytotoxicity, resulting in the elimination of the cancer (Wang et al., 2019). The structure of CARs consists of three main components—an external moiety that binds to the tumor antigen, a membrane spanning region, and an internal domain that induces T-cell activation (Muller et al., 2021). The outer segment typically uses a single-chain variable fragment (scFv) derived from an antibody that confers specificity (Krokhotin et al., 2019). The internal segment carries a CD3ζ domain required for T-cell activation and costimulatory domains such as CD28 or 4-1BB to enhance the activation signal (Boucher et al., 2021).

CAR T-cell therapy has shown impressive results in blood cancers, particularly B-cell acute lymphoblastic leukemia (B-ALL) and diffuse large B-cell lymphoma (DLBCL), with treatments such as Kymriah and Yescarta gaining FDA approval (Ma et al., 2019; Safarzadeh Kozani et al., 2021b). However, the efficacy of therapy in solid tumors is hampered by factors such as metastasis, tumor diversity, and the immunosuppressive tumor environment (Zhao et al., 2020b). Research is currently exploring potential solid tumor targets such as GD2 in neuroblastoma and human epidermal growth factor receptor 2 (HER2) in sarcoma (Straathof et al., 2020). To enhance the efficacy of CAR T-cell therapy in solid tumors, strategies such as regional administration and CARs that are immune to suppressive signaling are being considered (Huang et al., 2020).

Despite its great potential, CAR T-cell therapy has been observed to induce cytokine release syndrome (CRS) in more than 90% of patients and neurotoxicity due to overactivation in about half of the cases (Napolitano et al., 2022). CRS is often treated with drugs such as tocilizumab (Kauer et al., 2020). Ongoing efforts are directed at integrating suicide genes into CARs to improve their safety (Shi et al., 2020).

The future looks promising with the development of off-the-shelf universal CAR T-cells, multi-antigen targeting CARs, and the use of engineered Notch receptors to provide spatial control of CAR T-cell activation (Qu et al., 2019). Advances in CAR T-cell therapy could change the way cancer is treated. The ability to target solid tumors and improve the safety of treatments is a particularly exciting prospect in the evolution of this therapy.

#### 3.2.2 Tumor-targeting antibodies

Tumor-targeting antibodies are an integral part of immune-based cancer therapies. Their function depends on their ability to distinguish antigens that are differentially expressed on cancer cells, thus distinguishing these malignancies from healthy tissues. This leads to selective targeting and a reduction in untargeted or “off-target” effects (Hintz et al., 2021).

Over the past few decades, a number of these tumor-targeting antibodies have been approved. These developments have greatly improved the health outcomes of cancer patients (Sun et al., 2018). Rituximab, for example, targets CD20 on B cells, which are used to cure leukemia and lymphoma (Jain et al., 2021). Trastuzumab, on the other hand, reacts with HER2, which is overexpressed in about one-quarter to one-third of gastric and breast cancers and shows increased survival rates (Upton et al., 2021). Another example, cetuximab, blocks the epidermal growth factor receptor (EGFR), which plays a role in metastasis and growth, and has shown efficacy in colorectal and head and neck cancers (Dougherty et al., 2020; Trivedi and Ferris, 2021).

The most common mechanisms of anti-tumor action include blocking signaling pathways essential for cancer survival and proliferation. These mechanisms also recruit natural killer cells to enable antibody-dependent cellular cytotoxicity (ADCC). In addition, these mechanisms activate the complement system to enable complement-dependent cytotoxicity (CDC) (Murray et al., 2014; Zhu et al., 2020). Another effect of this mechanism is that the binding of the Fc region to cells of the immune system results in the stimulation of the immune defense against the cancerous tumor (Sasaki et al., 2020).

Nevertheless, resistance is observed in about half of all patients, often allowing relapse after an initial response to treatment (Licciardone and Aryal, 2014). This resistance can result from several mechanisms, including loss of expression of the target antigen, blockade of the antibody’s access to the cancer, and activation of other survival pathways (Fernandes et al., 2021). Another mechanism of resistance is through mutations in targets that allow evasion of antibodies (da Silva, 2012). Notably, even targeted therapies may have adverse effects due to non-specific distribution (Swati, 2021).

Current research is focused on a few areas to address these issues and further expand clinical utility. These areas include antibodies that are bispecific or multispecific, which can bind to more than one antigen as a method of not only improving specificity but also overcoming resistance (Ahamadi-Fesharaki et al., 2019). Another similar area of research is the study of drug–antibody conjugates, which use antibodies to selectively deliver chemotherapeutic agents or toxins (Pettinato, 2021). Another area of research is to increase the activation of the immune system through Fc engineering (van Tetering et al., 2020).

In sum, antibodies have had a significant impact on cancer care, leading to improved outcomes in certain malignancies. Therefore, it is highly likely that the future will see an expansion of antibodies as strategic, selective tools in therapy that harnesses the immune system to fight cancer.

#### 3.2.3 Checkpoint inhibitors

Checkpoint inhibitors have significantly changed the landscape of cancer immunotherapy through their ability to interfere with the immune evasion mechanisms employed by tumors (Zhang et al., 2021a). Tumor cells have developed sophisticated strategies to evade the initial defenses of the immune system or to actively suppress anti-cancer immunity mechanisms that have received considerable attention in cancer research (Mahata et al., 2020).

Critical regulatory roles in modulating normal immune responses and maintaining immune self-tolerance are played by checkpoint proteins such as programmed cell death protein 1 (PD-1), programmed death ligand 1 (PD-L1), and cytotoxic T-lymphocyte-associated antigen 4 (CTLA-4). PD-1, which is typically expressed on T-cells, binds to PD-L1 on healthy cells, and plays a regulatory role in preventing excessive T-cell activity (Schutz et al., 2017; Chun et al., 2022). Paradoxically, the same mechanism is used by tumor cells to evade T-cell attack by presenting PD-L1 on their surface (Hinterleitner et al., 2021). The introduction of PD-1 inhibitors interrupts this interaction, reviving the suppressed T cells and enabling them to identify and destroy tumor cells. This phenomenon has been demonstrated in the treatment of several cancers, including but not limited to melanoma and lung cancer (Curnock et al., 2021).

Also worthy of mention in the catalog of immune checkpoints is CTLA-4, which acts to inhibit T-cell activation during later stages of the immune response (Arra et al., 2021). Drugs such as ipilimumab are designed to target CTLA-4 and enhance anti-tumor immunity by removing this block to T-cell activation (Soldevilla et al., 2019). Clinical trials have demonstrated the efficacy of CTLA-4 inhibitors in the treatment of cancers, including melanoma (Hirshoren et al., 2020).

However, checkpoint inhibitors are not without limitations. More than half of treated patients experience immune-related adverse events (irAEs), the severity of which can range from mild to severe and in extreme cases can be life-threatening (Usui et al., 2022). Diarrhea, dermatitis, and hepatitis are among the most common irAEs (Spain et al., 2016). It is also noteworthy that some tumors are “cold” or non-inflamed, characterized by sparse immune cell infiltration into the tumor microenvironment. Patients with such tumors are less likely to respond to checkpoint inhibitor therapy (Gerard et al., 2021).

The scientific community is actively investigating strategies to overcome these challenges. Combination therapies using checkpoint inhibitors alongside other therapies such as chemotherapy, radiation, or targeted therapy are of great interest (Andersson and Ostheimer, 2019). Approaches aimed at making tumors more susceptible (or “hotter”) to immunotherapy are also under investigation, including the use of tumor antigen vaccines and optimized drug delivery approaches (Liu et al., 2020). Research is also underway to identify biomarkers that predict therapeutic response and risk of side effects (Bartsch et al., 2007).

In summary, although checkpoint inhibitors are revolutionizing cancer treatment through their ability to harness anti-tumor immunity, which is typically limited by immune evasion mechanisms employed by tumor cells, their efficacy is not universal, and side effects remain a notable concern. Current research efforts in combinatorial therapeutic strategies and predictive biomarker identification are aimed at improving clinical outcomes while minimizing toxic effects. Despite their limitations, checkpoint inhibitors show significant potential to improve cancer treatment. The challenge for the future is to extend the durability and safety of therapeutic responses, which will require continued research and innovation in the field.

## 4 Gene editing technologies and their potential in pediatric cancer treatment

Gene modification technologies have advanced significantly and have become critical in the study of cancer, particularly in pediatric oncology (West and Gronvall, 2020). In this field, the CRISPR system has proven to be particularly effective (Pomella and Rota, 2020). The CRISPR-Cas9 system allows scientists to precisely edit genetic sequences, a concept borrowed from bacterial immune defenses that precisely edit DNA (Neil et al., 2021). The Cas9 enzyme has been likened to molecular scissors that use RNA as a guide to specific genomic locations. This precision has led to unprecedented opportunities in pediatric cancer treatment (Smith et al., 2021).

The process of cancer development often involves the mutation or inactivation of certain genes, causing cells to multiply uncontrollably. Pediatric cancers are known for their aggressiveness and require prompt treatment. The correct application of CRISPR-Cas9 allows for the precise modification of defective genes, effectively halting or eliminating the growth of cancer cells (Oberlick et al., 2019; Dharia et al., 2021). Evidence from preclinical studies has underscored the potential of CRISPR-Cas9 to treat several types of pediatric cancer. For example, in neuroblastoma, CRISPR has been used to silence the MYCN oncogene, which is typically dysregulated in high-risk cases, resulting in a demonstrable anti-tumor effect (Yin et al., 2020). In osteosarcoma models, the application of CRISPR led to a reduction in tumor growth by targeting overactive TGF-β signaling (Hu et al., 2018).

However, ensuring a safe, targeted delivery mechanism to cancer cells while sparing healthy tissues remains a challenge and warrants further research (Rizwanullah et al., 2021). Approaches such as viral vectors are being explored to improve cell type specificity (Levy et al., 2020). In addition, the ethical issues associated with gene editing cannot be understated. The indelible nature of genetic modifications requires careful consideration of unintended off-target effects and long-term consequences, such as permanent changes in the germ line (Feeney, 2019).

In conclusion, while there is reason for optimism, in-depth clinical trials are essential to confirm the efficacy and safe application of CRISPR gene editing for pediatric cancers. Precision and accuracy of editing, coupled with minimal risks, are critical considerations before this technology is applied in clinical settings. Despite the clear challenges, the potential of this technology to revolutionize cancer treatment is profound.

## 5 Combination therapies involving gene therapy for enhanced efficacy

Gene therapy aims to treat diseases at their genetic root (Cicalese and Aiuti, 2020). However, refining and perfecting gene therapy remains a challenge. Research is underway to determine where gene therapy can be combined with other treatments to increase efficacy (Nastiuk and Krolewski, 2016). One promising approach has been to combine gene therapy with pharmacological or biological agents, especially in the case of complex genetic diseases (Rex, 2015). Improved outcomes have been reported in lysosomal storage disorders where gene therapy has been combined with enzyme replacement therapy (Li, 2018). Oncology researchers are investigating the use of gene therapy in combination with chemotherapy or immunotherapy to improve response (Chada et al., 2022). The logic of this approach is to augment the benefits of gene therapy with other treatment modalities (Lin et al., 2021). The combination of therapies may result in synergistic effects that exceed the effects of individual treatments, especially in diseases with both environmental and genetic contributions (Gaikani et al., 2021). Advanced gene editing techniques, such as CRISPR, and gene silencing methods, including RNA interference, are enabling innovations in combination therapies by allowing direct modification of gene sequence or expression (Zhang et al., 2021b). A comprehensive approach that integrates these methods with gene therapy may improve outcomes compared to any single therapy (Mac Gabhann et al., 2010). However, comprehensive studies are needed to ensure safety and long-term effects. Potential risks include off-target effects, unintended immune responses, and the need for lifelong monitoring (Kelley et al., 2002). In addition, research is needed to determine optimal dosing, timing, and patient selection to maximize benefits and minimize side effects (Ngoune et al., 2018).

In summary, strategies involving gene therapy represent a promising avenue for the treatment of complex diseases if existing challenges can be overcome. Despite the tremendous potential, ensuring safety and efficacy remains of paramount importance as these therapies continue to be refined and optimized. The future holds great promise for combination gene therapies, but careful and comprehensive research is essential to successfully translate this promise from theory to clinical practice.

## 6 Clinical applications and success stories in pediatric cancer

### 6.1 Overview of gene therapy clinical trials in pediatric cancer

Gene therapy is recognized as an important experimental approach to the treatment of pediatric cancers, the leading cause of disease-related death in children (Bornhorst and Hwang, 2016). Table 3 shows several clinical trials of gene therapies for pediatric cancer. Clinical trials are essential for evaluating efficacy and safety prior to clinical use (Jacobson et al., 2021). These trials typically progress through three phases, each with increasing numbers of participants, to methodically study efficacy and adverse effects in larger populations (Gore, 2003). For example, phase I trials confirm safety in a small cohort, phase II trials expand the cohort to evaluate efficacy, and phase III trials compare a new treatment with standard therapies using large cohorts (Millen and Yap, 2021).

**TABLE 3:** Clinical trials of gene therapies for pediatric cancer.

Trial identifier	Cancer type	Gene therapy	Gene therapy strategy	Combination therapy	Phase	Status	Clinical trial	Reference
NCT00634231	Pediatric brain tumors, including GBM, anaplastic astrocytoma, and recurrent ependymomas	Adv-TK	Viral vector (adenovirus)	Valacyclovir + radiation	I	Active	A Phase I study of AdV-tk + prodrug therapy in combination with radiation therapy for pediatric brain tumors	Kieran et al. (2019)
NCT03330197	Pediatric brain tumors or diffuse intrinsic pontine glioma (DIPG)	Ad-RTS-Hil-12	Viral vector (adenovirus)	Veledimex	I/II	Active	A study of Ad-RTS-Hil-12+veledimex in pediatric subjects with brain tumors or DIPG	RTS (2023)
NCT02457845	Pediatric recurrent or refractory cerebellar brain tumors	Oncolytic HSV-1	Viral vector (HSV-1)	Radiotherapy	I	Recruiting	HSV G207 in children with recurrent or refractory cerebellar brain tumors	Friedman et al. (2018)
NCT02435849	B-cell ALL	Anti-CD19	Anti-CD19 CAR T-cell	Tisagenlecleucel + tocilizumab	II	Completed	A single infusion of tisagenlecleucel provided durable remission with long-term persistence in pediatric and young adult patients with relapsed or refractory B-cell ALL, with transient high-grade toxic effects	Maude et al. (2018)
NCT03284268	Retinoblastoma	Oncolytic adenovirus	Viral vector (adenovirus)	VCN-01	I	Recruiting	Intravitreal administration of VCN-01 resulted in anti-tumor activity in retinoblastoma vitreous granules	Pascual-Pasto et al. (2019)
NCT03618381	Wilms tumor	EGFR	EGFR806 CAR-T cell	EGFR806 CAR T-cell + cetuximab/trastuzumab	I	Recruiting	Genetically modified to express an EGFR-specific receptor (chimeric antigen receptor or CAR) that will target and kill solid tumors that express EGFR and the selection suicide marker EGFRt	Albert et al. (2022)

Extensive preclinical studies providing proof of concept and confirming an appropriate therapeutic index provide a layer of safety before human trials begin. A prime example is CRISPR-Cas9, which allows precise editing of disease-causing mutations using a guide RNA to direct the Cas9 enzyme to desired DNA sequences (Suresh, 2021). Optimization of gene delivery methods is also part of preclinical work using animal models and cell culture experiments (Bez et al., 2019).

Early phase studies have already demonstrated the potential success of gene therapy in non-cancer diseases, including sickle cell disease, inherited retinal diseases, HIV, and transthyretin amyloidosis (Grimley et al., 2020). Clinical improvements in disease markers and symptom manifestations in patients suggest considerable potential for pediatric cancers as well (Delforge et al., 2022).

Despite these promising advances, risks such as off-target effects, where gene modifications inadvertently alter other genomic sites, warrant attention. An unintended consequence could be the onset of cancer if genes that suppress tumors are silenced (Peltomaki, 2012). Another potential risk is immunogenicity, or the induction of an immune response against the introduced genetic material (Koyama et al., 2009). The long-term effects of permanent changes in the genome are still unclear and undefined (Long et al., 2011). Trial participants must be fully informed that there is no guarantee of benefit and that unforeseen risks may occur (Dube et al., 2017). Intensive research efforts are essential to develop the promising initial results before gene therapy can be widely applied to childhood cancers (Khan and Helman, 2016). Key priorities include optimizing patient selection, delivery methods, dosing, and management of side effects. Further advances in technology and thorough scientific investigation are essential to ultimately realize the rich potential of gene therapy for the benefit of children with cancer.

### 6.2 Successful cases of gene therapy in pediatric cancer treatment

Gene therapy, an innovative form of treatment that works by altering the genes within an individual’s cells, has shown considerable therapeutic potential, particularly in pediatric malignancies (Handgretinger and Schlegel, 2018). The process involves addressing and correcting abnormalities caused by mutations, thereby restoring the normal function of proteins that may be dysfunctional or missing (Handgretinger and Schlegel, 2018). Several implementations of this approach have been successful in pediatric oncology, as described below.

A strategy known as gene addition is proving effective against diseases that arise from a single gene. A functional variant of an underperforming gene is introduced into the system using specially programmed viral or non-viral vectors. As a result, the introduced gene compensates for the deficiency by producing the previously missing proteins (Zeng et al., 2021). Examples of remarkable effects of this method can be seen in the treatment of neuroblastoma, where the administration of a gene-encoded antibody via an adenoviral vector significantly reduced tumor size in the majority of patients in clinical trials (Chen et al., 2022). The gene-addition strategy has also been successful in ameliorating the effects of X-linked severe combined immunodeficiency, a disorder that renders individuals susceptible to infectious diseases (Blanco et al., 2020).

An alternative treatment method, post-transcriptional gene silencing, works by neutralizing specific messenger RNAs to prevent expression of the affected gene. This approach has been used to knock out the MYCN oncogene in high-risk neuroblastoma, significantly slowing cancer cell growth (Mendez et al., 2015). Targeting and silencing ALK oncogenic mutations with this method also significantly reduced tumor progression in neuroblastoma models (Durand et al., 2019).

In addition, gene editing, which involves the modification, deletion, or addition of DNA, is another tool in the application of gene therapy to pediatric malignancies (Zhang et al., 2021b). Of note in this category is CAR T-cell therapy, which showed efficacy when tested in pediatric leukemia (Greinix, 2019). Promising results have also been observed in preliminary studies when PD-1 was edited to induce T cells to attack osteosarcoma tumors (Fan et al., 2021).

The mechanics of each of these gene therapy platforms rely heavily on the delivery of genetic material via a compendium of engineered viral or non-viral vectors—a critical component of the entire operation (Bulcha et al., 2021). The examples presented highlight the monumental progress that has been made to date in the treatment of pediatric cancers with gene therapy. However, further exploration of the long-term implications is warranted, and a comprehensive risk-benefit analysis should be conducted in consultation with health care professionals. As research continues to improve the efficacy of gene therapy, we cannot overlook the significant potential this approach brings to the field of pediatric cancer treatment.

### 6.3 Challenges and limitations encountered in clinical applications

Clinical applications of gene therapy for pediatric cancers have made progress but still face significant challenges that limit widespread adoption and success (Xia et al., 2020).

One major challenge stems from the complexity of pediatric tumors. Unlike adult cancers that accumulate mutations, pediatric malignancies often arise from developmental defects that compromise the efficacy of gene therapy (Jessa et al., 2019; Blattner-Johnson et al., 2022). For example, pediatric brain tumors such as medulloblastoma have different underlying biology and growth patterns than adult tumors, making them less amenable to certain gene therapy approaches. In addition, determining the optimal dosage, timing, and delivery method poses significant hurdles (Li and Langhans, 2021). Careful fine-tuning is essential to maximize tumor destruction while minimizing toxicity and side effects, requiring intensive monitoring (Pasquier et al., 2019). In a trial for recurrent high-grade glioma, some patients experienced brain inflammation and neurologic toxicity, which were attributed to difficulties with dose calibration (Chiocca et al., 2019).

Of great concern are potential long-term unintended consequences. The introduction of genetic modifications risks “off-target” effects on genes other than the intended target, potentially triggering problems such as secondary cancers (Nelson et al., 2019). Tragically, X-linked severe combined immune deficiency (X-SCID) gene therapy trials inadvertently caused leukemia in some patients due to activation of the LMO2 oncogene (Ruggero et al., 2016).

In addition, patients’ immune systems may attack the introduced genes, reducing efficacy and jeopardizing health (Ledford and Callaway, 2018). In an X-SCID trial, some patients developed an immune response that attacked their own blood cells (Ehl et al., 2005). Other barriers include complex regulations, exorbitant manufacturing/distribution costs, and specialized administration (Ehl et al., 2005). For example, it took more than 20 years from the first cancer gene therapy trials to FDA approval of CAR T-cell therapy in 2017 (Libutti, 2019).

**FIGURE 2:**
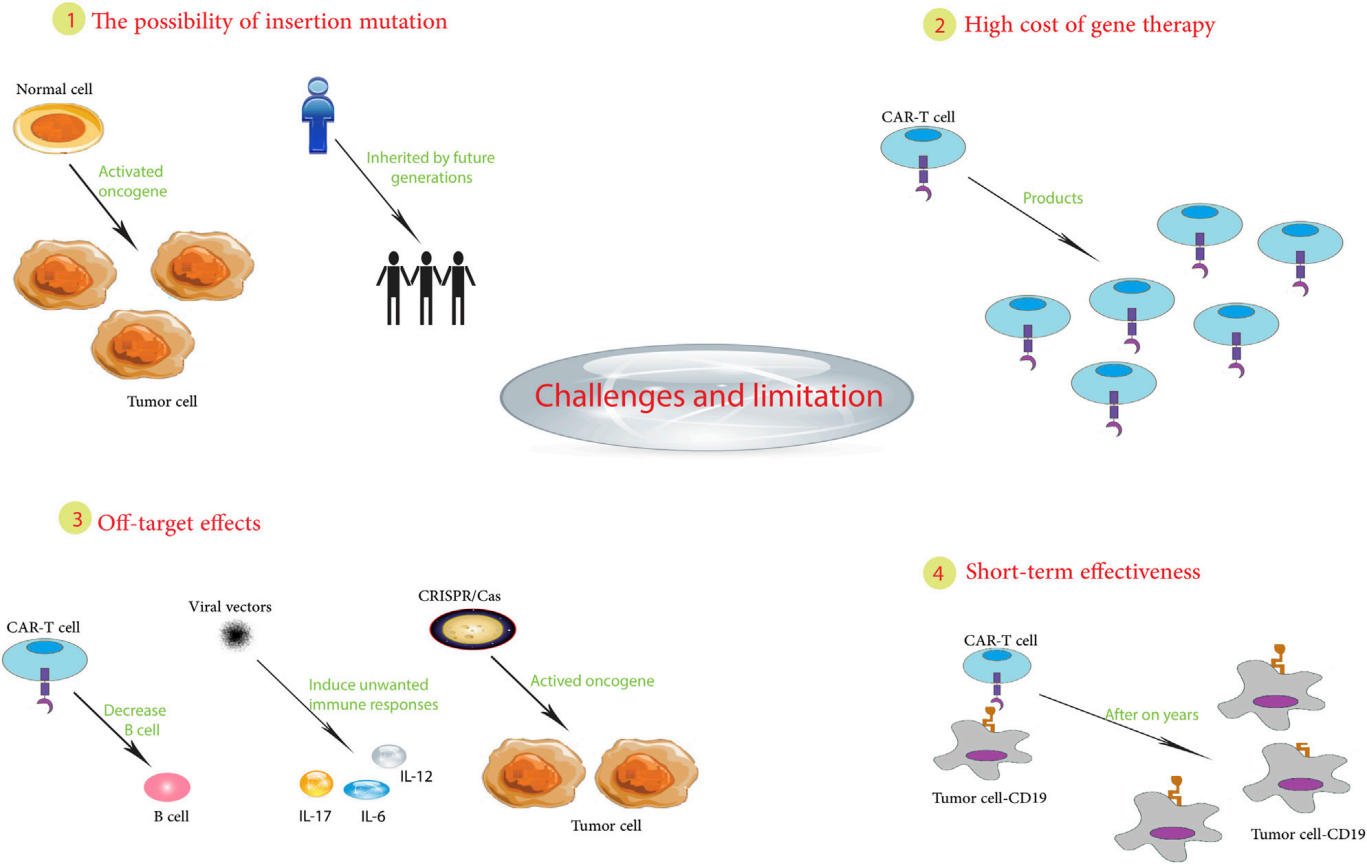
Challenges and limitations in pediatric cancer gene therapy.

In summary, pediatric cancer gene therapy still faces challenges, including tumor biology, treatment optimization, unintended effects, immune responses, regulations, and implementation (Figure 2). Continued innovation in vectors, delivery, and precision oncology may help overcome these barriers and realize the promise of gene therapy for children with cancer. While hurdles remain, the possibilities make it a worthwhile pursuit.

## 7 Potential risks and adverse effects associated with gene therapy in pediatric patients

Although gene therapy holds great promise for the treatment of childhood cancers, it is associated with various risks and adverse effects that must be carefully evaluated. A key hurdle is the precise manipulation of genes to target disease without causing unintended harmful side effects (Wedekind et al., 2018).

Significant risks are associated with the vectors used to deliver therapeutically important genes into cells. Despite their regular use, even modified viral vectors are known to induce unwanted immune responses, leading to inflammatory or allergic reactions. A clinical trial of adenoviral vectors for ornithine transcarbamylase deficiency resulted in the unfortunate death of a pediatric patient due to a severe immunologic reaction to the viral carrier (Brown et al., 2017). There is also the potential for off-target effects. While the goal is to replace defective genes, unintended alteration of other genes may occur. Such unintended changes can have potentially adverse health consequences, including the development of secondary cancers. For example, in a trial for X-linked severe combined immunodeficiency, gene therapy inadvertently activated the LMO2 proto-oncogene, leading to leukemia in some patients (Qasim and Gkazi, 2019).

In addition, the irreversible nature of gene modification can result in adverse effects that may not manifest until later in a patient’s life. Particularly in pediatric cases, predicting long-term outcomes is complex (Baum et al., 2003). For example, in animal models, gene therapy used to treat mucopolysaccharidosis has been associated with the later onset of neurological and skeletal abnormalities (Zapolnik and Pyrkosz, 2021). There is also a risk of insertional mutagenesis, a process in which newly inserted genetic material disrupts other genes. Integration of viral vectors could induce mutations by activating oncogenes, potentially causing cancer. In clinical trials of X-linked chronic granulomatous disease, vector insertion appeared to induce malignant transformation in some patients (Knight et al., 2013; Ha et al., 2021).

In addition, risks have been identified related to germline effects, where genetic alterations in pediatric patients could be inherited by future generations. This issue raises significant ethical concerns, particularly in the case of non-life-threatening diseases (Rubeis and Steger, 2018). The implementation of strict safeguards around vector delivery to target tissues is critical to prevent unintended germline transmission. From an ethical perspective, the relatively novel nature of gene therapy, coupled with permanent genetic alterations, requires careful consideration in obtaining informed consent in pediatric patients to protect vulnerable pediatric patients and their families from potential exploitation (Rubeis and Steger, 2018).

In summary, risks such as immune reactions, off-target effects, long-term effects, insertional mutagenesis, germline transmission, and ethical dilemmas must be addressed to realize the full potential of gene therapy in pediatric cancer. Ongoing research is aimed at optimizing vectors, improving precision delivery, and monitoring long-term effects to reduce risks and safely extend the benefits of gene therapy to more pediatric patients. Although there are risks involved, the judicious use of gene therapy offers a glimmer of hope for many children who have previously faced a shortened life expectancy.

## 8 Conclusion

Pediatric oncology is poised for revolutionary advances due to the significant potential of gene therapy. Ongoing research efforts to unravel the genetics of pediatric cancer have made it increasingly important to foster interdisciplinary collaboration to improve gene therapy applications. Concurrent initiatives encourage collaboration among clinicians, researchers, and advocates to accelerate the clinical translation process.

Gene therapy offers extraordinary potential to transform pediatric cancer treatment by facilitating targeted, personalized therapeutic strategies that minimize toxicity to healthy cells. Its efficacy has been validated by early successes, demonstrating the possibility of a durable and precise treatment era. However, realizing the full potential of gene therapy will require overcoming hurdles related to delivery, safety, policy, and social inequalities and will require a concerted focus on strategic innovation and reform. Equally critical is the management of expectations, as the clinical translation of many emerging gene therapies remains a distant reality.

Looking ahead, the field appears poised for accelerated progress, driven by scientific innovation and cross-sector collaboration. The role of gene therapy is expected to expand and significantly contribute to improving pediatric cancer survival rates while enhancing quality of life. Maximizing the benefits will require continued research, advocacy, and policy advances to catalyze the transformative journey from the laboratory to the clinic. Underpinned by the patient-centered core principle, the future appears hopeful and filled with opportunities to translate the tremendous promise of gene therapy into accessible treatments for pediatric patients worldwide.

## Abbreviation

CAR, chimeric antigen receptor; ALL, acute lymphoblastic leukemia; CDKN, cyclin-dependent kinase inhibitor; CRISPR, clustered regularly interspaced short palindromic repeats; SCID, severe combined immune deficiency; ADA, adenosine deaminase; GOSH, Great Ormond Street Hospital; GVHD, graft-vs-host disease; RNAi, RNA interference; ALK, anaplastic lymphoma kinase; miRNAs, microRNAs; AAVs, adeno-associated viruses; LNPs, lipid nanoparticles; HIV, human immunodeficiency virus; scFv, single-chain variable fragment; B-ALL, B-cell acute lymphoblastic leukemia; CRS, cytokine release syndrome; HER2, human epidermal growth factor receptor 2; EGFR, epidermal growth factor receptor; ADCC, antibody-dependent cellular cytotoxicity; CDC, complement-dependent cytotoxicity; PD-1, programmed cell death protein 1; PD-L1, programmed death ligand 1; CTLA-4, cytotoxic T-lymphocyte-associated antigen 4; irAEs, immune-related adverse events.
